# Effect of Penetration Enhancers on Transdermal Delivery of Oxcarbazepine, an Antiepileptic Drug Using Microemulsions

**DOI:** 10.3390/pharmaceutics15010183

**Published:** 2023-01-04

**Authors:** Amitkumar Virani, Vinam Puri, Hana Mohd, Bozena Michniak-Kohn

**Affiliations:** 1 Ernest Mario School of Pharmacy, Rutgers-The State University of New Jersey, 160 Frelinghuysen Road, Piscataway, NJ 08854, USA; 2 Center for Dermal Research, Rutgers-The State University of New Jersey, 145 Bevier Road, Piscataway, NJ 08854, USA

**Keywords:** oxcarbazepine, epilepsy, transdermal, penetration enhancer, permeation, microemulsion

## Abstract

Oxcarbazepine (OXC) is an anticonvulsant drug, indicated for the treatment of the neurological disorder, epilepsy. The objective of the present study was to evaluate the transdermal delivery of OXC from microemulsions using different penetration enhancers. Transcutol^®^ P (TRC), oleic acid (OA), cineole (cin), Labrasol (LS), Tween 80 (T80) and *N*-Methyl-Pyrrolidone (NMP) were used as penetration enhancers as well as microemulsion components. Simple formulations of OXC in propylene glycol (PG) incorporating various penetration enhancers and combination of penetration enhancers were also evaluated for transdermal delivery. Drug delivery and penetration enhancement were studied using human cadaver skin on Franz diffusion cells. The results showed that all penetration enhancers improved the rate of permeation of OXC compared to the control. The flux of drug delivery from the various formulations was found to be, in decreasing order, cin > OA + TRC > NMP > TRC > OA. Overall, microemulsions prepared using cineole, Tween 80 and Transcutol^®^ P (TRC) were shown to be provide the best penetration enhancement for OXC.

## 1. Introduction

Epilepsy is a chronic neurological disorder reported in people of all ages and it is marked by recurrent seizures [1]. The cause of these recurrent seizures is the abnormal discharge of neurons within the central nervous system (CNS). Oxcarbazepine (OXC) is an antiepileptic drug, utilized primarily in the treatment of epilepsy since it controls many types of seizures. In the United States, OXC was approved by Food and Drug Administration (FDA) as an anticonvulsant for the treatment of epilepsy in 2002. OXC is approved as a monotherapy for the treatment of partial seizures in adults with epilepsy and for the adjunctive treatment of partial seizures in children aged 4–16 years old [2,3]. Its mechanism of action is reported to be the stabilization of the neuron membrane and reduction in seizures by blocking sodium uptake channels. According to the Biopharmaceutical Classification System (BCS), OXC is categorized as a Class II drug, meaning it possesses low water solubility but high permeability through membranes of the body [4]. It has a water solubility of 308 mg/L, a partition coefficient of 1.5 (Log P) and a molecular weight of 252.27 g/mole [5].

Currently, oxcarbazepine tablet and suspension formulations are commercially available and both formulations were developed and manufactured by Novartis Pharmaceuticals under the brand name of Trileptal^®^. Supernus Pharmaceuticals, Inc. (Rockville, MD, USA), a specialty pharmaceutical company, developed Oxtellar XR^®^ as a prolonged release tablet of oxcarbazepine [6]. Antiepileptic drugs are usually available as either tablets and/or suspensions for oral administration. Oral administration can be an inconvenient route of administration in some situations such as for stroke patients who are not able to swallow, are vomiting and/or for unconscious patients, as well as for elderly patients with dysphagia (swallowing difficulties). The maximum oral dose limit is 5 mL for children aged below 4 years and 10 mL for children aged 4–12 years old. The taste of the drug is the major issue for pediatric and psychiatric patients who do not want to take the formulations [7,8]. Transdermal drug delivery systems (TDDS) can be considered as an alternative route of administration to overcome some, if not all, of these issues. Various formulations have been evaluated for OXC delivery, such as microemulsions for intranasal delivery, PLGA (Poly Lactic-co-Glycolic Acid) nanoparticles for topical drug delivery, modified release interpenetrating polymer network (IPN) macromolecule beads and oxcarbazepine-loaded nanoparticles for treating pregnant women with epilepsy [4,9,10,11].

Transdermal drug delivery has gained considerable attention because this route offers several advantages over other more conventional routes of drug delivery [12,13]. Since the drug is delivered through skin layers it avoids pre-systemic metabolism by the liver and gastrointestinal tract degradation associated with oral drug delivery [14,15]. Ease of application and flexibility of termination of drug administration by removing transdermal delivery patch from skin are additional advantages. This delivery system is also suitable for pediatric, geriatric, psychiatric, unconscious and vomiting patients [14,16,17]. Currently, only about twenty drugs are available for transdermal patch drug delivery because exceptionally few drugs have the required physicochemical properties to penetrate through the skin, which provides a significant barrier against drug absorption through skin [18,19].

Chemical penetration enhancers increase the drug transport across the skin by reducing the skin barrier. They are also known as permeation enhancers. Adding permeation enhancers to the formulation to temporarily and reversibly raise the stratum corneum’s permeability is one of the most common techniques to enhance the performance of transdermal formulations. Overcoming the stratum corneum barrier allows for the effective and safe transport of medications through the skin [20]. Over the years, various chemical classes and their components, including alcohols, essential oils, azones, chelating agents, and surfactants, have been investigated [21]. Permeation enhancers increase the penetration of drug molecules by three mechanisms of action. Firstly, they promote lipid extraction from the skin. Secondly, they interrupt the lipids’ order in the stratum corneum and hence increase the fluidity and decrease the resistance to solutes. Lastly, they increase the drug diffusion by enhancing the partition parameter through the skin [21,22,23]. Cineole and oleic acid were selected as penetration enhancers, as they act as an oil phase in the preparation of microemulsion. Transcutol^®^ P and *N*-Methyl-Pyrrolidone were selected as penetration enhancers as well as cosurfactants in microemulsion preparation.

Microemulsions are thermodynamically stable colloidal dispersions composed of oil, water and surfactant or a combination of surfactant and co-surfactant. They are clear, low viscous, monophasic, optically isotropic liquids with small droplet sizes [24]. There are several advantages of using microemulsions for transdermal delivery since they can increase the permeation of both hydrophobic and hydrophilic drugs by increasing their solubility and enabling the creation of a favorable concentration gradient into skin. Microemulsions with a smaller droplet size have also been reported to have increased success in skin permeation [25,26]. 

In this study, we investigated the effect of various penetration enhancers on the transdermal delivery of oxcarbazepine. Microemulsions were then prepared using these penetration enhancers and evaluated for in-vitro skin permeation. The long-term goal of our study is to develop new oxcarbazepine formulations for the transdermal route of administration to treat epilepsy. 

## 2. Materials and Methods

### 2.1. Materials 

OXC was purchased from Euticals Spa, Italy and used as the drug molecule. Cineole (cin), *N*-methyl-Pyrrolidone (NMP), oleic acid (OA) and Transcutol^®^ P (TRC) were investigated as penetration enhancers. OA was a gift from Croda Inc., Edison, NJ, USA. TRC and Labrasol (LS) were gifts from Gattefosse Corporation, Paramus, NJ, USA. Cin, NMP, HPLC grade methanol and water, Propylene Glycol (PG), Polysorbate 80 (Tween 80), Isopropyl Myristate (IPM), Medium Chain Triglyceride (MCT), Polyethylene Glycol 400 (PEG-400), Sodium Lauryl Sulfate (SLS), Triethanolamine (TEA) and formic acid were obtained from Sigma-Aldrich, Saint Louis, MO, USA. Carbopol 980 was a gift from Lubrizol, Wickliffe, OH, USA. Ethanol was procured from Decon Labs, Inc., King of Prussia, PA, USA. Phosphate-Buffered Saline (PBS) was prepared by dissolving one PBS tablet in 100 mL of water. Add SLS to PBS at 5% and pH was adjusted to 5.8. PBS tablets were purchased from MP Biomedicals, Solon, OH, USA. Dermatomed human cadaver skin from the posterior torso region of 59-year-old male was used for the ex-vivo permeation study and was supplied by New York Firefighter Skin Bank, New York, NY, USA.

### 2.2. Methods 

#### 2.2.1. Solubility Determinations

Solubility determination of OXC was performed using glass vials filled with 10 mL of PG, IPM, MCT, PEG-400, methanol, ethanol and PBS alone or with 5–15% of penetration enhancers OA, TRC, cin and NMP. An excess amount of OXC was added to create saturated suspension. The suspensions were vigorously mixed by vortexing the vials for 3 min, followed by sonication in a water bath for 60 min. The samples were then agitated at 25 °C for 48 h using a shaker to reach equilibrium between the saturated solution and undissolved drug. The resulting suspensions were passed through 0.2 µm polypropylene syringe filter to remove undissolved OXC. The amount of dissolved OXC in the filtrate was further diluted and quantified by using HPLC. 

#### 2.2.2. High-Performance Liquid Chromatography (HPLC)

The assay methodology utilized an Agilent 1100 series high-performance liquid chromatograph (HPLC) coupled with UV detection (with diode array detector—DAD) and Agilent Chemstation software (OpenLab CDS, Chemstation Edition, Rev. C.01.10, Agilent Technologies, Santa Clara, CA, USA). A Phenomenex C18 column (150 mm × 4.6 mm, 5.0 µ particle size) was used as the stationary phase at 25 °C (Phenomenex, Torrance, CA, USA). The mobile phase methanol: water (with 0.02% formic acid) was used in a 50:50 ratio by volume at a flow rate of 1.0 mL/min with the sample injection volume of 20 μL with a run time of 10 min. The retention time for OXC was 6.1 min with UV detection at a wavelength of 229 nm. Linearity of the peak area vs. concentration was recorded with a standard concentration range from 0.25 µg/mL to 100 µg/mL and the coefficient of regression (R^2^) of 0.99 was obtained. The % RSD for intra-day and inter-day precision of the method was 0.1% and 0.9%, respectively. The limit of detection was 0.25 µg/mL and limit of quantification was 0.5 µg/mL.

#### 2.2.3. In Vitro Skin Permeation Study of OXC

The permeation study was carried out using vertical glass Franz diffusion cells (Logan Instruments, Somerset, NJ, USA). Dermatomed human cadaver skin from the posterior torso of a 59-year-old male donor was supplied by New York Firefighter Skin Bank (New York, NY, USA) and stored in a freezer (−80 °C) until use. The skin was quickly thawed in pH 7.4 PBS at room temperature for 5 min. After thawing the skin, it was cut into approximately 2 cm^2^ pieces and hydrated in PBS pH 7.4 for 15 min before the permeation study. Each skin piece was mounted between the donor and receptor chambers with the stratum corneum facing upward toward the donor chamber and lower part of skin in contact with receptor chamber with a diffusion area of 0.64 cm^2^ [27]. The receptor chamber was filled with 5.0 mL of PBS + 5% SLS (pH 5.8), stirred continuously at 600 rpm using a magnetic stirrer, maintained at 37 °C ± 0.5 °C to keep skin temperature at 32 °C ± 0.5 °C and was allowed to equilibrate with skin for 15 min for applying the dose [28]. The donor phase contained 0.2 mL of suspension of OXC in PG vehicle alone or with 5% *w/w* of penetration enhancers. Samples of 300 µL were taken from the sampling arm of the Franz cells at 3 h, 6 h, 12 h, 18 h, 21 h and 24 h, followed by replenishment with fresh receptor media. All the samples were analyzed using the validated HPLC method mentioned above. 

#### 2.2.4. Preparation of Microemulsions

##### Phase Diagrams

The water titration method was used to construct ternary phase diagrams and is described below [29]. The ternary phase diagram consists of oil phase, surfactant phase (mixture of surfactant and co-surfactant) and water. A fixed ratio of surfactant to co-surfactant (1:1) by weight was prepared for phase diagram. Each oil phase was mixed with surfactant phase in the glass vials at ratios of 1:1, 1:2, 1:3, 1:4, 1:5, 1:6, 1:7, 1:8 and 1:9. These mixtures were mixed with a vortex mixer (Fisherbrand™ Analog Vortex Mixer, Hampton, NH, USA) before adding ultrapure water, which was used as water phase to titrate these mixtures and mixed again with the vortexer. Titration was continued until the mixture turned turbid from transparent and the mixtures were concluded as microemulsions until turning turbid. For each microemulsion system, the percentages of the oil phase, surfactant phase and water phase were calculated to construct ternary phase diagrams. 

##### Placebo (No Drug) Microemulsion Preparation

In the first step of microemulsion preparation, oil phase was mixed with surfactant phase, then water was added and mixed it. Cin and OA were used as oil phase, LS and Tween 80 (T80) as surfactants and TRC and PEG 400 as co-surfactants, and ultrapure water was used for water phase. The purpose of this placebo microemulsion preparation was to select microemulsion with smaller droplet size for drug loading. The compositions of placebo microemulsions are described in Table 1. 

##### Drug-Loaded Microemulsions

In the first step of drug-loaded microemulsion preparation, the oil phase was mixed with surfactant and the co-surfactant phase. Next, drug was added and mixed it until it dissolved. Finally, water was added and mixed. The compositions of drug-loaded microemulsions are described in Table 2. 

##### Drug-Loaded Microemulsion Gel Formulation

First, the drug-loaded microemulsion was prepared, and then 0.1% (*w*/*w*) of Carbopol 980 was added and mixed well. The final gel was prepared by neutralizing the Carbopol 980 using triethanolamine to final pH 6.5 ± 0.5. 

#### 2.2.5. Characterization of the Microemulsions

##### pH

The pH value of the microemulsion was determined by using a pH meter (Fisherbrand™ Accumet™ AP115 Portable pH Meter Kit). The pH meter was calibrated before use by using pH standard calibration solutions (Sigma-Aldrich, Inc., USA). 

##### Viscosity

The viscosity of the microemulsion was determined by using the Brookfield DV3TLV Rheometer (AMETEK Brookfield, MA, USA). Viscosity was measured by using spindle SC18 at 30 RPM and the set-up temperature was 23 °C ± 1 °C.

##### Refractive Index

The refractive index (RI) of the microemulsion was measured by using the Refractometer (Cole-Parmer Digital Refractometer, 0–95% Brix, 1.3330–1.5400 RI).

##### Visual Observation

All the microemulsions were observed visually after preparation and they appeared transparent.

##### Droplet Size and Polydispersity Index

The droplet size and polydispersity index (PDI) of the microemulsion was measured by using the Zetasizer (Malvern Zetasizer Nano ZS90). 

#### 2.2.6. Data Analysis

The transdermal permeation of OXC over 24 h from different formulations was quantified using the validated HPLC assay. The permeation profile was plotted by drawing graph of cumulative amount of drug permeated through a unit area of skin as a function of time. The flux (J) value was calculated as a slope of the linear portion of plot. The average OXC flux was calculated by performing individual experiments three times. Permeability coefficient (P) and enhancement ratio (ER) were calculated using following equations [27]:

P = J (Flux)/C (OXC concentration in donor)

ER = Flux with the enhancer (test formulation)/Flux without enhancer (control formulation)

One-way ANOVA and Student’s *t*-test were used to treat the experiment data with the threshold for significance of the data (*p*-value) being less than or equal to 0.05.

## 3. Results and Discussion

### 3.1. Solubility Study 

The saturation solubility of OXC in different vehicles alone or with 5% *w*/*w* of penetration enhancers, as determined using HPLC, have been reported in Table 3 and Table 4. The results show that the highest solubility was obtained with PEG-400 (9.70 ± 0.64 mg/mL). The lowest solubility was found in the case of MCT (0.20 ± 0.01 mg/mL) and IPM (0.20 ± 0.01 mg/mL). The addition of penetration enhancers increased the solubility of OXC in PG. The highest solubility (5.90 ± 0.05 mg/mL) was obtained with 20% of cin in PG. The solubility of OXC in PBS pH 5.8 (0.10 ± 0.01 mg/mL). SLS was added to the receptor media to increase the solubility of OXC. The solubility of OXC in PBS pH 5.8 was increased to 1.60 ± 0.12 mg/mL and 2.50 ± 0.14 mg/mL by adding 3% SLS and 5% SLS, respectively. 

### 3.2. Effect of Penetration Enhancers

**Figure 1:**
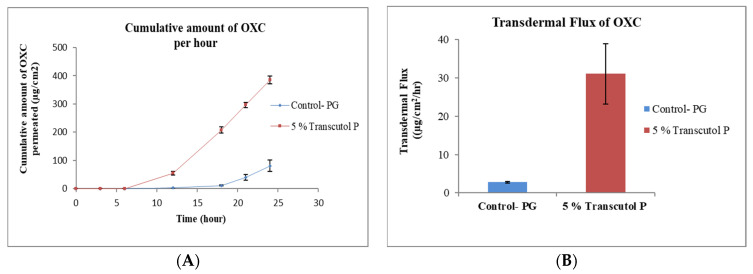
In vitro cumulative amount of OXC permeated per unit area versus time (**A**) and transdermal flux of OXC (**B**) across the human cadaver skin in presence or absence of Transcutol^®^ P. Each point represents the mean ± S.D. of three replicates in the absence of multiple donors.

**Figure 2:**
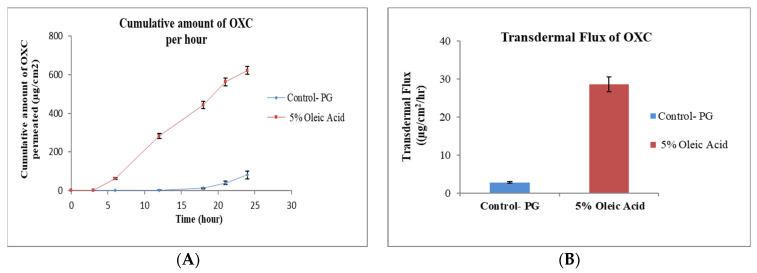
In vitro cumulative amount of OXC permeated per unit area versus time (**A**) and transdermal flux of OXC (**B**) across the human cadaver skin in presence or absence of oleic acid. Each point represents the mean ± S.D. of three replicates in the absence of multiple donors.

**Figure 3:**
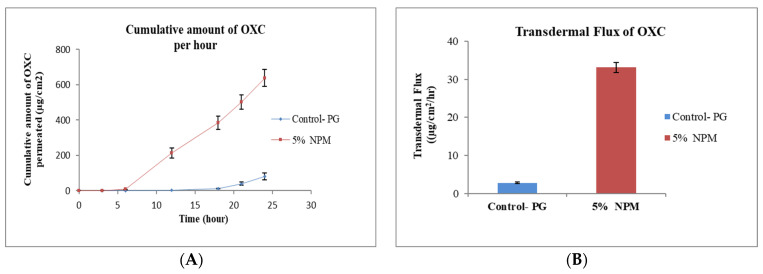
In vitro cumulative amount of OXC permeated per unit area versus time (**A**) and transdermal flux of OXC (**B**) across the human cadaver skin in presence or absence of *N*-Methyl 2-Pyrrolidone. Each point represents the mean ± S.D. of three replicates in the absence of multiple donors.

**Figure 4:**
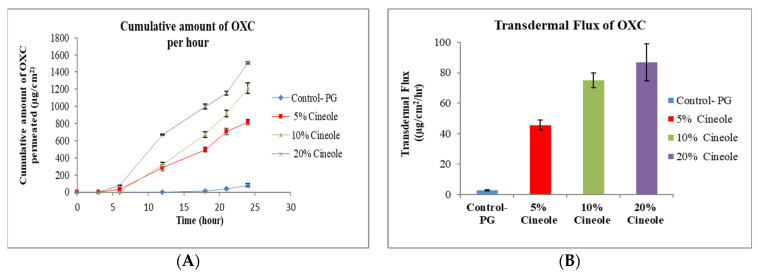
In vitro cumulative amount of OXC permeated per unit area versus time (**A**) and transdermal flux of OXC (**B**) across the human cadaver skin in presence or absence of cineole. Each point represents the mean ± S.D. of three replicates in the absence of multiple donors.

**Figure 5:**
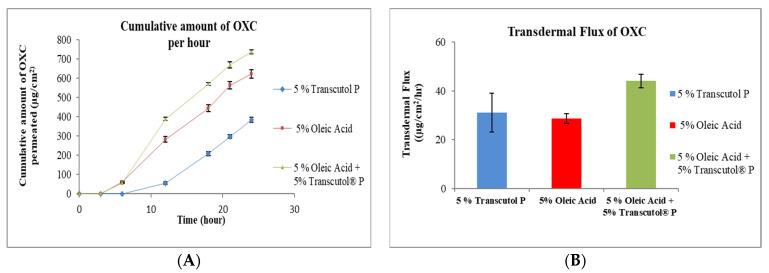
In vitro cumulative amount of OXC permeated per unit area versus time (**A**) and transdermal flux of OXC (**B**) across the human cadaver skin in presence or absence of the combination of Transcutol^®^ P and oleic acid. Each point represents the mean ± S.D. of three replicates in the absence of multiple donors.

PG is a commonly used glycol and solvent for many lipophilic drugs [30]. A simple formulation of OXC in PG was used as control sample. The effect permeation profile of OXC from different formulation containing penetration enhancers TRC, OA, cin and NMP are shown in Figure 1, Figure 2, Figure 3, Figure 4 and Figure 5. The different formulations of OXC with varying permeation enhancers were applied to the human cadaver skin and the permeation experiments were performed for 24 h. It was found that the addition of the penetration enhancer significantly increases (*p* < 0.05) the skin permeation rate of OXC in the order of cin > OA + TRC > NMP > TRC > OA.

#### 3.2.1. Effect of Transcutol^®^ P

Transcutol (diethylene glycol monoethyl ether) is a clear (transparent) and hygroscopic liquid. It is chemically an ethylene oxide derivative and non-toxic [31]. TRC is soluble in water and miscible with non-polar solvents, making it strong solubilizer for hydrophilic and hydrophobic drug molecules [31]. TRC is soluble in PG, glycerin, ethanol and water, and miscible with MCT and PG [32]. TRC is currently used as solvent in over 500 cosmetic products and in the first FDA-approved prescription formulation for acne, Aczone^®^ (dapsone) topical gel 5% [33]. TRC increases the penetration of drug molecules by “pull” effect [34]. Pull effect is explained by the dragging of the drug molecules while diffusing through skin. Skin accumulation of genistein and flux was increased by the utilizing TRC in topical gel. The enhancement of genistein permeation was by the formation of the “intracutaneous depot’’ by modifying the stratum corneum and increasing the solubility of the drug by using TRC in topical gel [35]. Diclofenac, clonazepam and minoxidil are drug molecules that have been shown to effectively penetrate the skin using TRC for topical drug delivery [36,37,38]. The results from the present experiment demonstrate that the permeation of OXC from TRC formulation was increased against the control formulation without TRC (Figure 1A). The flux of OXC increased from 2.90 ± 0.22 μg/cm^2^/h for formulation without enhancer to 31.10 ± 7.93 μg/cm^2^/h for formulation with 5% TRC as shown in Figure 1B. 

#### 3.2.2. Effect of Oleic Acid

Oleic acid has been found to enhance the permeation of several drugs [39,40]. OA increases the permeation of lipophilic drugs through the skin by the transdermal cellular pathway. The mechanism of action for OA is the enhancement of permeant diffusion through the skin by disordering skin lipids in the stratum corneum (SC). OA interacts with lipid chains of the SC ceramides and the disturbed arrangement leads to the lipid fluidity [41]. OA has been shown to enhance the transdermal delivery of donepezil base through human cadaver and mouse skin [39]. The permeation of Aspirin was increased 7-fold by utilizing OA as an enhancer in a gel formulation [42]. The permeation enhancement of thymoquinone was concluded by using OA at concentration 5% in formulation against the control formulation [28]. The results from the present experiment demonstrate that the permeation of OXC from OA formulation increased against the control formulation without OA (Figure 2A). The flux of OXC increased from 2.90 ± 0.22 μg/cm^2^/h for formulation without enhancer to 28.60 ± 2.01 μg/cm^2^/h for formulation with 5% OA as shown in Figure 2B.

#### 3.2.3. Effect of *N*-Methyl Pyrrolidone

NMP has significantly gained interest in the pharmaceutical industry in recent years as a transdermal enhancer. The permeation of Estradiol was increased by incorporating 10% NMP in the oily gel formulation [43]. NMP is a polar solvent and easily mixed with water. It acts by interacting with the polar head of lipids, which changes the lipid structure and loosens lipid packaging in the SC. It increases the drug mobility into skin, yielding a higher flux of the permeant [44]. The transdermal penetration of dimethyl fumarate increased 2.5-fold by utilizing NMP at 20% concentration [27]. The results from our investigation demonstrate that the permeation of OXC from NMP formulation increased against the formulation without an enhancer (Figure 3A). The flux of OXC increased from 2.90 ± 0.22 μg/cm^2^/h for the control formulation without enhancer to 33.10 ± 1.32 μg/cm^2^/h for formulation with 5% NMP as shown in Figure 3B.

#### 3.2.4. Effect of Cineole

Cineole is the most extensively studied terpene for permeation enhancement of drugs in skin. Cin possesses a polar group which provides an increased penetration enhancement for hydrophilic drug molecules. The mechanism action of terpenes is interaction with SC intercellular lipids to facilitate permeation [45]. The permeation enhancement study of lidocaine and ofloxacin from the moisture-activated transdermal patches by using terpenes such as cin and limonene showed that the patch containing cin was able to enhance the permeation of both drugs, and it was better than l-menthol and d-limonene [46]. It was found that cin can promote the skin penetration of mefenamic acid by “pull” or “drag” effect, where both enhancer and drug molecule simultaneously penetrate through skin [47]. Low boiling point terpenes can easily interact with SC lipids due to weak cohesiveness, and thereby enhance drug penetration. The permeation study of zidovudine concluded that cin with a lower boiling point (173 °C) possessed better permeation enhancement than that of carvone with a higher boiling point (230 °C) [48]. Cin was also able to increase the solubility and permeation of dimethyl fumarate [27]. The current results demonstrate that the permeation of OXC from the cin formulation increased against the control formulation without cin (Figure 4A). The addition of a higher amount of cin in the formulation improved the permeation of OXC. The flux of OXC increased from 2.90 ± 0.22 μg/cm^2^/h for formulation without enhancer to 45.60 ± 3.22 μg/cm^2^/h, 74.90 ± 5.00 μg/cm^2^/h, 86.90 ± 12.10 μg/cm^2^/h for formulation with 5% cineole, 10% cineole and 20% cineole, respectively, as shown in Figure 4B.

#### 3.2.5. Combination of Transcutol^®^ P and Oleic Acid

Figure 5 shows that OXC formulation with combination of 5% of TRC and 5% OA significantly increased the flux of OXC. It was observed that the flux of OXC from the formulation with a combination of penetration enhancers (44.00 ± 2.80 μg/cm^2^/h) was 1.5-fold higher compared with OA alone (28.60 ± 2.01 μg/cm^2^/h) and 1.4-fold higher compared with TRC alone (31.10 ± 7.93 μg/cm^2^/h). Such action of TRC and OA in the formulation can be explained based on the increment of the drug solubility in the formulation, and there may also be a synergistic effect produced by the combination of TRC and OA. A study conducted by Aboofazeli, R., H. Zia, and T.E. Needham concluded that mixture of PG, OA, and dimethyl isosorbide (80:10:10 *v*/*v*) showed synergistic interaction among the formulation components and yielded maximum flux of nicardipine hydrochloride [49].

#### 3.2.6. Transdermal Flux of Oxcarbazepine

**Figure 6:**
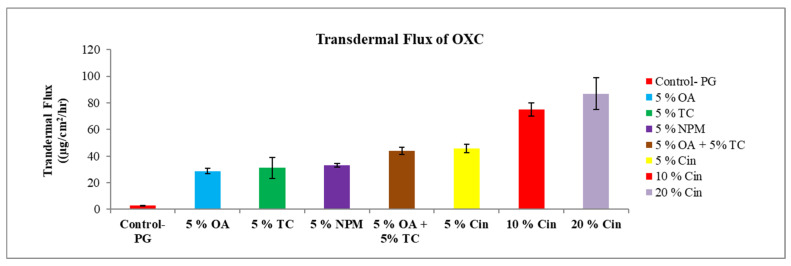
Transdermal flux of OXC across human cadaver skin in the presence or absence of different penetration enhancers. Each point represents the mean ± S.D. of three replicates in the absence of multiple donors.

The flux of OXC from the various formulations in presence of penetration enhancer was found to be in decreasing order as cin > OA + TRC > NMP > TRC > OA. Furthermore, increase in cineole concentration in formulation resulted in improvement in the flux of oxcarbazepine, as shown in Figure 6. Oxcarbazepine appeared to penetrate through human skin in vitro by using different penetration enhancers.

### 3.3. Pseudoternary Phase Diagrams

**Figure 7:**
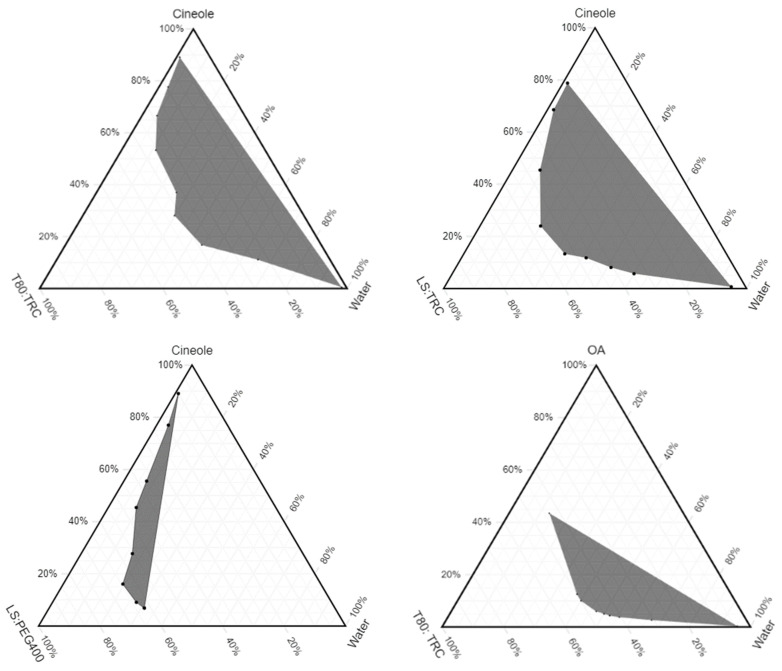

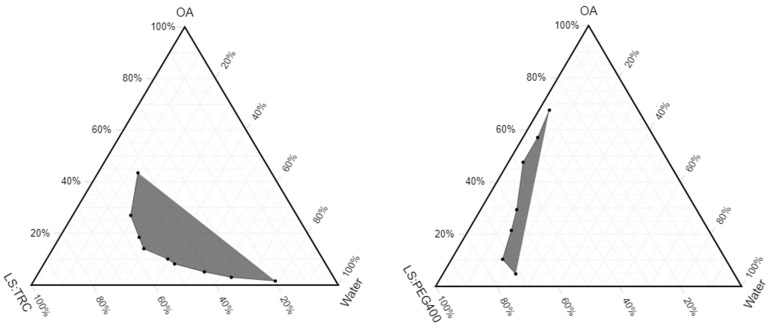
Pseudoternary phase diagrams of microemulsions formulated using oil (cin and OA), surfactant (T80 and LS), co-surfactant (PEG 400 and TRC), and water. Surfactant to co-surfactant ratio is 1:1.

A pseudoternary phase diagram provides a region in the mixture of the three phases where microemulsions exist. The ternary phase diagram was prepared by using OA, cin, T80, LS, TRC and PEG 400 (Figure 7). The ratio of surfactant to co-surfactant was 1:1 for all the microemulsions. The shaded area in the ternary phase diagram corresponds to transparent mixtures, which is identified as microemulsions, while the white area depicts turbid mixtures. The phase diagram clearly indicates that with a combination of T80 and LS, and T80 and TRC, a larger area of microemulsion is created for both the oils cin and OA compared to the combination of PEG 400 and LS. 

### 3.4. Microemulsion

The use of permeation enhancers in the transdermal delivery of several drugs has been widely studied. Spaglova et al. used the microemulsion system to deliver minoxidil transdermally using natural permeation enhancers [50]. Another recent study used oleic acid as a permeation enhancer to deliver nifedipine, an antihypertensive drug [51]. Other publications in the literature have studied transdermal permeation using microemulsion systems using different or combinations of permeation enhancers [52]. A nanoemulsion prepared using oleic acid and eucalyptol (cineol) as a penetration enhancer increased the permeation of caffeine and naproxen compared to the control sample without penetration enhancers [53]. This work demonstrated that microemulsion prepared using oleic acid or cineole significantly enhanced OXC permeation as compared to control sample.

Initially, placebo microemulsions ME1 to ME12 were prepared. These microemulsions were tested for droplet size, pH, RI, PDI and viscosity (Table 5). It appears that microemulsions prepared with LS and PEG 400 as surfactant and co-surfactant have a higher droplet size (135.20 ± 3.55 nm–272.90 ± 4.97 nm) than other microemulsions (35.24 ± 1.31 nm–156.90 ± 5.46 nm). The microemulsions with a droplet size less than 200 nm were selected for permeation study. 

There is no significant change in droplet size after adding drugs into microemulsion than corresponding placebo microemulsions (Table 6). The microemulsions show droplet sizes ranging from 31.44 ± 2.35 nm to 161.40 ± 10.03 nm. The PDI of all microemulsions were less than 1.0, indicating that all the prepared microemulsions were homogeneous. The pH range of the microemulsions were observed to be from 5.13 to 6.84, which is close to the human skin pH range and is not expected to irritate the skin. The viscosities of the microemulsions ranged from 17.20 cP to 33.20 cP. 

**Figure 8:**
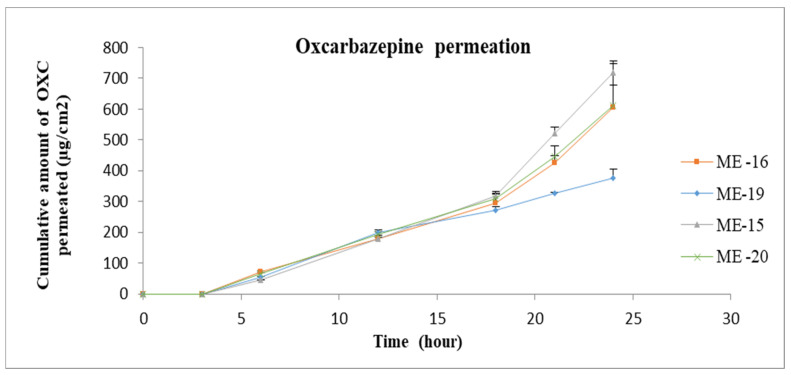
In vitro cumulative amount of OXC permeated per unit area versus time across the human cadaver skin. Each point represents the mean ± S.D. of three replicates in the absence of multiple donors. ME15-10%OA, 30%T80, 30%TRC, 30%Water. ME16-10%OA, 30%TRC, 30%LS, 30%Water. ME19-5%OA, 25%T80, 25%TRC, 45%Water. ME16-5%OA, 25%TRC, 25%LS, 45%Water.

**Figure 9:**
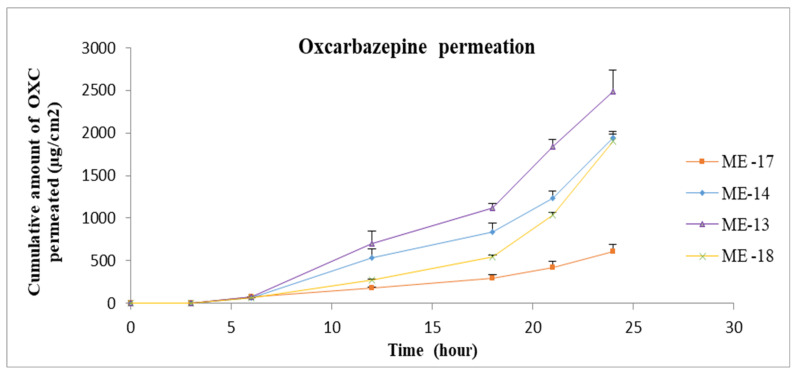
In vitro cumulative amount of OXC permeated per unit area versus time across the human cadaver skin. Each point represents the mean ± S.D. of three replicates in the absence of multiple donors. ME13-10%Cin, 25%T80, 25%TRC, 40%Water. ME14-10%Cin, 30%TRC, 30%LS, 30%Water. ME17-5%Cin, 20%T80, 20%TRC, 55%Water. ME18-5%Cin, 25%TRC, 25%LS, 45%Water.

**Figure 10:**
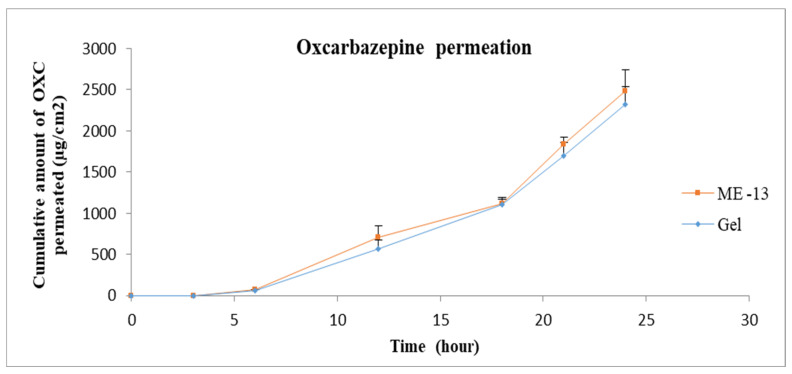
In vitro cumulative amount of OXC permeated per unit area versus time across the human cadaver skin. Each point represents the mean ± S.D. of three replicates in the absence of multiple donors. ME13-10%Cin, 25%T80, 25%TRC, 40%Water.

**Figure 11:**
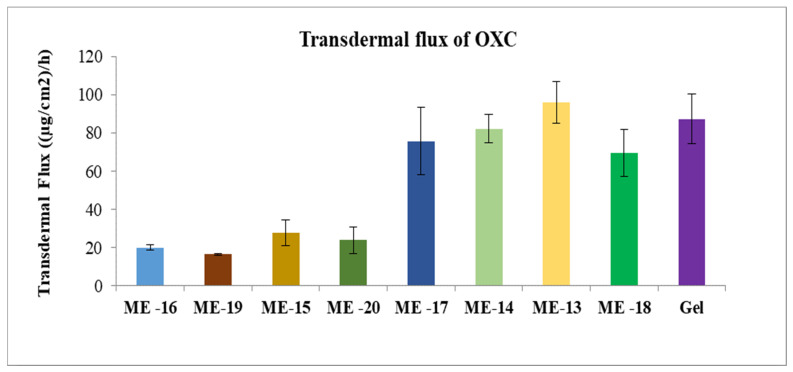
Transdermal flux of OXC across the human cadaver skin from microemulsions. Each point represents the mean ± S.D. of three replicates in the absence of multiple donors.

**Figure 12:**
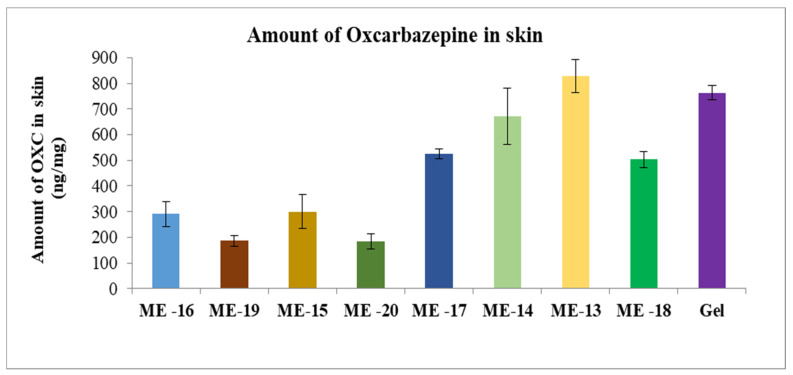
Amount of OXC deposited in the skin after 24 h of experiment. Each point represents the mean ± S.D. of three replicates in the absence of multiple donors.

The gel formulation was prepared by adding Carbomer 980 to ME 13. The permeation profiles of oxcarbazepine from microemulsions and gel formation through skin are shown in Figure 8, Figure 9 and Figure 10. It was clear from the permeation data that the microemulsions prepared by using cineole as oil (ME-13,14,17,18) have higher permeation than the microemulsions prepared by using oleic acid as oil (ME-15,16,19,20). The flux of OXC from the various formulations sown in Figure 11. The amount of oxcarbazepine present in skin after 24 h was detected as shown in Figure 12. Among all the formulations, the microemulsion ME-13 exhibited highest permeation flux and drug permeation.

There are other works in the literature that have investigated the transdermal permeation using microemulsion system using different permeation enhancers or combination of permeation enhancers. However, to the best of our knowledge this is the only work that studies the permeation of OXC transdermally. Furthermore, this research study evaluates the impact of different permeation enhancer as well as the combination of permeation enhancers to deliver OXC transdermally. Overall, Transcutol^®^ P, oleic acid, cineole and N-Methyl-Pyrrolidone have significantly modified the permeation of OXC. However, skin irritation, cytotoxicity study and in-vivo study can be conducted.

## 4. Conclusions

The permeation and flux of OXC across the human cadaver skin were significantly higher in presence of various penetration enhancers in the formulations containing PG as the main solvent. Transcutol^®^ P, oleic acid, cineole and *N*-Methyl-Pyrrolidone have significantly modified the permeation of OXC. Cineole at 20% *w*/*w* in PG was found to increase the flux of OXC by 30-fold. Use of a combination of penetration enhancers showed synergistic effects. Transcutol and oleic acid at 5% *w*/*w* in PG increased the flux of OXC by 15.17-fold. This research showed that Transcutol^®^ P, oleic acid, cineole and *N*-Methyl-Pyrrolidone will be valuable to develop an alternative route of administration of OXC to treat neurological disorders such as epilepsy, and the findings can be used to enhance such developments in the future.

## Figures and Tables

**Table 1:** Composition of placebo microemulsions used in the study.

% *w*/*w*							
	Cin	OA	T80	TRC	LS	PEG 400	Water
ME 1	10	0	25	25	0	0	40
ME 2	10	0	0	30	30	0	30
ME 3	10	0	0	0	40	40	10
ME 4	0	10	30	30	0	0	30
ME 5	0	10	0	30	30	0	30
ME 6	0	10	0	0	40	40	10
ME 7	5	0	20	20	0	0	55
ME 8	5	0	0	25	25	0	45
ME 9	5	0	0	0	35	35	25
ME 10	0	5	25	25	0	0	45
ME 11	0	5	0	25	25	0	45
ME 12	0	5	0	0	40	40	15

ME: microemulsion; cin: cineole; OA: oleic acid; T80: Tween 80; TRC: Transcutol^®^ P; LB: Labrasol; PEG-400: Polyethylene Glycol 400.

**Table 2:** Composition of microemulsions 13–20.

% *w*/*w* Oxcarbazepine (0.6%)							
	Cin	OA	T80	TRC	LS	PEG 400	Water
ME 13	10	0	25	25	0	0	40
ME 14	10	0	0	30	30	0	30
ME 15	0	10	30	30	0	0	30
ME 16	0	10	0	30	30	0	30
ME 17	5	0	20	20	0	0	55
ME 18	5	0	0	25	25	0	45
ME 19	0	5	25	25	0	0	45
ME 20	0	5	0	25	25	0	45

ME: microemulsion; cin: cineole; OA: oleic acid; TRC: Transcutol^®^ P; LB: Labrasol; PEG-400: Polyethylene Glycol 400; T80: Tween 80.

**Table 3:** Summary of the solubility of OXC at 48 h (mean ± S.D.) in the tested vehicles expressed as mg/mL (n = 3).

Solvent	Solubility mg/mL
Propylene Glycol	2.90 ± 0.05
Isopropyl Myristate	0.20 ± 0.01
Medium Chain Triglycerides	0.20 ± 0.01
Polyethylene Glycol 400	9.7 0± 0.64
Tween 80	4.40 ± 0.98
Methanol	3.50 ± 0.41
Ethanol	1.40 ± 0.14
Phosphate Saline Buffer (pH 5.8)	0.10 ± 0.01
Phosphate Saline Buffer (pH 5.8) + 1% SLS	0.60 ± 0.10
Phosphate Saline Buffer (pH 5.8) + 3% SLS	1.60 ± 0.12
Phosphate Saline Buffer (pH 5.8) + 5% SLS	2.50 ± 0.14

**Table 4:** Summary of the solubility and permeation parameters of OXC through human cadaver skin in tested formulations in presence or absence of penetration enhancers (n = 3).

Formulation	Solubility (mg/mL) ± SD	OXC Flux (µg/cm²/h)	OXC Q_24_ (µg/cm²)	P × 10^−3^ (cm/h) ± SD	ER ^b^
Control	2.90 ± 0.05	2.90 ± 0.22	81.00 ± 18.91	0.28 ± 0.02	1
5% Oleic Acid	3.30 ± 0.30	28.60 ± 2.01 ^a^	621.90 ± 20.72	3.08 ± 0.77	9.86
5% Transcutol	3.30 ± 0.07	31.10 ± 7.93 ^a^	385.40 ± 13.44	4.49 ± 0.28	10.72
5% *N*-Methyl-2-pyrrolidone	3.70 ± 0.19	33.10 ± 1.32 ^a^	638.70 ± 47.41	2.84 ± 0.19	11.41
5% Oleic Acid + 5% Transcutol	3.80 ± 0.01	44.00 ± 2.80 ^a^	736.40 ± 11.01	3.28 ± 0.11	15.17
5% Cineole	3.30 ± 0.18	45.60 ± 3.22 ^a^	816.40 ± 31.51	4.39 ± 0.31	15.72
10% Cineole	4.70 ± 0.01	74.90 ± 5.00 ^a^	1213.30 ± 60.32	7.42 ± 0.45	25.82
20% Cineole	5.90 ± 0.05	86.90 ± 12.10 ^a^	1506.60 ± 10.02	9.01 ± 0.94	29.97

^a^ The flux of OXC increased significantly (*p* < 0.05). ^b^ The flux of test formulation/The flux of control formulation.

**Table 5:** Droplet size, pH, RI, PDI and viscosity of placebo microemulsions.

Microemulsion	Mean Droplet Size (nm)	PDI	pH	Viscosity (cP)	RI
ME 1	38.70 ± 1.88	0.44 ± 0.056	4.41	32.50	1.41
ME 2	82.14 ± 2.18	0.21 ± 0.01	3.75	26.40	1.42
ME 3	203.40 ± 5.73	0.72 ± 0.02	6.37	60.20	1.45
ME 4	156.90 ± 5.46	0.41 ± 0.10	4.74	34.20	1.41
ME 5	91.13 ± 3.90	0.26 ± 0.02	4.23	27.40	1.40
ME 6	272.90 ± 4.97	0.12 ± 0.01	5.92	61.40	1.45
ME 7	26.96 ± 0.78	0.45 ± 0.18	4.10	18.50	1.38
ME 8	66.52 ± 1.05	0.20 ± 0.00	3.94	17.60	1.39
ME 9	193.60 ± 8.85	0.30 ± 0.07	6.37	45.80	1.42
ME 10	35.24 ± 1.31	0.64 ± 0.123	4.40	21.40	1.40
ME 11	87.26±1.38	0.22±0.02	4.82	19.20	1.39
ME 12	135.20±3.55	0.22±0.01	6.05	48.60	1.45

ME: microemulsion; PDI: polydispersity index; RI: refractive index; cP: centipois.

**Table 6:** Droplet size, pH, RI, PDI and viscosity of oxcarbazepine microemulsions.

% *w*/*w* Oxcarbazepine (0.6%)					
Microemulsion	Mean Droplet Size (nm)	PDI	pH	Viscosity (cP)	RI
ME 13	41.42 ± 3.42	0.37 ± 0.03	5.61	31.60	1.41
ME 14	94.13 ± 3.22	0.25 ± 0.03	6.84	27.10	1.41
ME 15	161.40 ± 10.03	0.54 ± 0.10	5.38	33.20	1.41
ME 16	109.70 ± 5.30	0.242 ± 0.05	5.42	28.10	1.41
ME 17	31.44 ± 2.35	0.40 ± 0.12	5.20	17.40	1.38
ME 18	68.28 ± 2.37	0.28 ± 0.04	6.14	16.90	1.38
ME 19	42.98 ± 1.40	0.60 ± 1.40	5.13	21.90	1.41
ME 20	87.24 ± 0.97	0.16 ± 0.02	5.86	17.20	1.39

ME: microemulsion; PDI: polydispersity index; RI: refractive index; cP: centipois.

## Data Availability

Not applicable.

## Abbreviations

OXC	Oxcarbazepine
TRC	Transcutol^®^ P
OA	Oleic Acid
Cin	Cineole
NMP	*N*-Methyl-Pyrrolidone
PG	Propylene Glycol
PBS	Phosphate Saline Buffer
LS	Labrasol
T80	Tween 80
PG	Propylene Glycol
IPM	Isopropyl Myristate
MCT	Medium Chain Triglyceride
MO	Mineral Oil
ME	Microemulsion (s)
TEA	Triethanolamine

## References

[1] Banerjee P.N., Filippi D., Hauser W.A. (2009). The descriptive epidemiology of epilepsy—A review. Epilepsy Res..

[2] Penny B., Ensom M.H.H. (2008). Does Oxcarbazepine Warrant Therapeutic Drug Monitoring?. Clin. Pharmacokinet..

[3] Mazza M., Della Marca G., Di Nicola M., Martinotti G., Pozzi G., Janiri L., Bria P., Mazza S. (2007). Oxcarbazepine improves mood in patients with epilepsy. Epilepsy Behav..

[4] Prajapati V.D., Gandhi A.K., Patel K.K., Patel B.N., Chaudhari A.M., Jani G.K. (2015). Development and optimization of modified release IPN macromolecules of oxcarbazepine using natural polymers. Int. J. Biol. Macromol..

[5] PubChemCID:34312. National Center for Biotechnology Information. PubChem Database. Oxcarbazepine CID = 34312. https://pubchem.ncbi.nlm.nih.gov/compound/Oxcarbazepine.

[6] Nam K., Ha E.-S., Kim J.-S., Kuk D.-H., Ha D.-H., Kim M.-S., Cho C.-W., Hwang S.-J. (2017). Solubility of oxcarbazepine in eight solvents within the temperature range T = (288.15–308.15) K. J. Chem. Thermodyn..

[7] Sastry S.V., Nyshadham J.R., Fix J.A. (2000). Recent technological advances in oral drug delivery–A review. Pharm. Sci. Tech. Today.

[8] Batchelor H.K., Marriott J.F. (2015). Formulations for children: Problems and solutions. Br. J. Clin. Pharmacol..

[9] Tirunagari M., Sameen J., Nandagopal A. (2017). Formulation Development and Characterization of Oxcarbazepine Microemulsion for Intranasal Delivery. Acta Pharm. Sci..

[10] Singh M.P., Kaushik A. (2012). Preparation and evaluation of plga nanocarriers gel for topical delivery. Der. Pharmacia. Lettre..

[11] Lopalco A., Ali H., Denora N., Rytting E. (2015). Oxcarbazepine-loaded polymeric nanoparticles: Development and permeability studies across in vitro models of the blood–brain barrier and human placental trophoblast. Int. J. Nanomed..

[12] Liuzzi R., Carciati A., Guido S., Caserta S. (2016). Transport efficiency in transdermal drug delivery: What is the role of fluid microstructure?. Colloids Surf B Biointerfaces.

[13] Roberts M.S., Cheruvu H.S., Mangion S.E., Alinaghi A., Benson H.A., Mohammed Y., Holmes A., van der Hoek J., Pastore M., Grice J.E. (2021). Topical drug delivery: History, percutaneous absorption, and product development. Adv. Drug Deliv. Rev..

[14] Schoellhammer C.M., Blankschtein D., Langer R. (2014). Skin permeabilization for transdermal drug delivery: Recent advances and future prospects. Expert Opin. Drug. Deliv..

[15] Marwah H., Garg T., Goyal A.K., Rath G. (2016). Permeation enhancer strategies in transdermal drug delivery. Drug. Deliv..

[16] Ita K. (2014). Transdermal drug delivery: Progress and challenges. J. Drug Deliv. Sci. Technol..

[17] Prausnitz M.R., Langer R. (2008). Transdermal drug delivery. Nat. Biotechnol..

[18] Moffatt K., Wang Y., Singh T.R.R., Donnelly R.F. (2017). Microneedles for enhanced transdermal and intraocular drug delivery. Curr. Opin. Pharmacol..

[19] Nguyen J., Ita K., Morra M., Popova I. (2016). The influence of solid microneedles on the transdermal delivery of selected antiepileptic drugs. Pharmaceutics.

[20] Ruan J., Liu C., Wang J., Zhong T., Quan P., Fang L. (2022). Efficacy and safety of permeation enhancers: A kinetic evaluation approach and molecular mechanism study in the skin. Int. J. Pharm..

[21] William A., Barry B. (2004). Penetration enhancer. Adv. Drug Deliv..

[22] Morteza-Semnani K., Saeedi M., Akbari J., Eghbali M., Babaei A., Hashemi S.M.H., Nokhodchi A. (2022). Development of a novel nanoemulgel formulation containing cumin essential oil as skin permeation enhancer. Drug Deliv. Transl. Res..

[23] Thong H.-Y., Zhai H., Maibach H.I. (2007). Percutaneous penetration enhancers: An overview. Ski. Pharmacol. Physiol..

[24] Tartaro G., Mateos H., Schirone D., Angelico R., Palazzo G. (2020). Microemulsion microstructure(s): A tutorial review. Nanomaterials.

[25] Yang J., Xu H., Wu S., Ju B., Zhu D., Yan Y., Wang M., Hu J. (2017). Preparation and evaluation of microemulsion-based transdermal delivery of Cistanche tubulosa phenylethanoid glycosides. Mol. Med. Rep..

[26] Callender S.P., Mathews J.A., Kobernyk K., Wettig S.D. (2017). Microemulsion utility in pharmaceuticals: Implications for multi-drug delivery. Int. J. Pharm..

[27] Ameen D., Michniak-Kohn B. (2017). Transdermal delivery of dimethyl fumarate for Alzheimer’s disease: Effect of penetration enhancers. Int. J. Pharm..

[28] Haq A., Michniak-Kohn B. (2018). Effects of solvents and penetration enhancers on transdermal delivery of thymoquinone: Permeability and skin deposition study. Drug Deliv..

[29] Das S., Lee S.H., Chia V.D., Chow P.S., Macbeath C., Liu Y., Shlieout G. (2020). Development of microemulsion based topical ivermectin formulations: Pre-formulation and formulation studies. Colloids Surf B Biointerfaces.

[30] Bendas B., Schmalfuβ U., Neubert R. (1995). Influence of propylene glycol as cosolvent on mechanisms of drug transport from hydrogels. Int. J. Pharm..

[31] Sullivan D.W., Gad S.C., Julien M. (2014). A review of the nonclinical safety of Transcutol®, a highly purified form of diethylene glycol monoethyl ether (DEGEE) used as a pharmaceutical excipient. Food Chem. Toxicol..

[32] Osborne D.W., Musakhanian J. (2018). Skin penetration and permeation properties of Transcutol^®^—Neat or diluted mixtures. AAPS PharmSciTech.

[33] Osborne D.W. (2011). Diethylene glycol monoethyl ether: An emerging solvent in topical dermatology products. J. Cosmet. Dermatol..

[34] Haque T., Talukder M.M.U. (2018). Chemical enhancer: A simplistic way to modulate barrier function of the stratum corneum. Adv. Pharm. Bull..

[35] Chadha G., Sathigari S., Parsons D.L., Babu J. (2011). In vitro percutaneous absorption of genistein from topical gels through human skin. Drug Dev. Ind. Pharm..

[36] Mura P., Faucci M., Bramanti G., Corti P. (2000). Evaluation of transcutol as a clonazepam transdermal permeation enhancer from hydrophilic gel formulations. Eur. J. Pharm. Sci..

[37] Mura S., Manconi M., Sinico C., Valenti D., Fadda A.M. (2009). Penetration enhancer-containing vesicles (PEVs) as carriers for cutaneous delivery of minoxidil. Int. J. Pharm..

[38] Manconi M., Mura S., Sinico C., Fadda A., Vila A., Molina F. (2009). Development and characterization of liposomes containing glycols as carriers for diclofenac. Colloids Surf. A Physicochem. Eng. Asp..

[39] Choi J., Choi M.-K., Chong S., Chung S.-J., Shim C.-K., Kim D.-D. (2012). Effect of fatty acids on the transdermal delivery of donepezil: In vitro and in vivo evaluation. Int. J. Pharm..

[40] Santoyo S., Arellano A., Ygartua P., Martin C. (1995). Penetration enhancer effects on the in vitro percutaneous absorption of piroxicam through rat skin. Int. J. Pharm..

[41] Lane M.E. (2013). Skin penetration enhancers. Int. J. Pharm..

[42] Ammar H.O., Ghorab M., El-Nahhas S.A., Kamel R. (2007). Evaluation of chemical penetration enhancers for transdermal delivery of aspirin. Asian J. Pharm. Sci..

[43] Koizumi A., Fujii M., Kondoh M., Watanabe Y. (2004). Effect of N-methyl-2-pyrrolidone on skin permeation of estradiol. Eur. J. Pharm. Biopharm..

[44] Barry B.W. (1987). Mode of action of penetration enhancers in human skin. J. Control. Release.

[45] Chen J., Jiang Q.-D., Chai Y.-P., Zhang H., Peng P., Yang X.-X. (2016). Natural terpenes as penetration enhancers for transdermal drug delivery. Molecules.

[46] Song Y.H., Gwak H.S., Chun I.K. (2009). The effects of terpenes on the permeation of lidocaine and ofloxacin from moisture-activated patches. Drug Deliv..

[47] Heard C.M., Kung D., Thomas C.P. (2006). Skin penetration enhancement of mefenamic acid by ethanol and 1, 8-cineole can be explained by the ‘pull’effect. Int. J. Pharm..

[48] Narishetty S.T.K., Panchagnula R. (2004). Transdermal delivery of zidovudine: Effect of terpenes and their mechanism of action. J. Control. Release.

[49] Aboofazeli R., Zia H., Needham T.E. (2002). Transdermal delivery of nicardipine: An approach to in vitro permeation enhancement. Drug Deliv..

[50] Špaglová M., Čuchorová M., Čierna M., Poništ S., Bauerová K. (2021). Microemulsions as solubilizers and penetration enhancers for minoxidil release from gels. Gels.

[51] de Araujo G.R.S., da Cruz Macieira G.M., de Oliveira D.X., Matos S.S., Dos Santos Q.N., Otubo L., de Souza Araújo A.A., Duarte M.C., Lira A.A.M., de Souza Nunes R. (2022). Microemulsions formed by PPG-5-CETETH-20 at low concentrations for transdermal delivery of nifedipine: Structural and in vitro study. Colloids Surf. B Biointerfaces.

[52] Song H., Liu C., Ruan J., Yang D., Zhong T., Liu Y., Fang L. (2022). Effect of the combination of permeation enhancer and ion-pairs strategies on transdermal delivery of tofacitinib. Int. J. Pharm..

[53] Abd E., Namjoshi S., Mohammed Y.H., Roberts M.S., Grice J.E. (2016). Synergistic skin penetration enhancer and nanoemulsion formulations promote the human epidermal permeation of caffeine and naproxen. J. Pharm. Sci..

